# Acute skin allergy to thermoplastic mask used for patient immobilization during radiation therapy: a case report

**DOI:** 10.1186/s13256-018-1715-y

**Published:** 2018-06-27

**Authors:** Nicolas Massager, Cécile Renier, Daniel Devriendt

**Affiliations:** 1 Department of Neurosurgery, University Hospital Tivoli, avenue Max Buset 34, 7100 La Louviere, Belgium; 2 Department of Radiophysics, Institut Bordet, boulevard de Waterloo 212, 1000 Brussels, Belgium; 3 Department of Radiation Therapy, Institut Bordet, boulevard de Waterloo 212, 1000 Brussels, Belgium

**Keywords:** Thermoplastic mask, Allergy, Radiation therapy, Gamma knife radiosurgery, Side effect

## Abstract

**Background:**

Radiosurgical treatments of brain tumors, vascular malformations, and functional disorders are more and more frequently used. Gamma Knife irradiation with the Icon system necessitates the use of a thermoplastic mask for head immobilization during treatment. Acute cutaneous allergy to thermoplastic masks has never been reported.

**Case presentation:**

A 71-year-old Caucasian woman treated radiosurgically for a sphenoidal meningioma using the Icon Gamma Knife system developed an acute allergic skin reaction to the thermoplastic mask used for head immobilization. Corticoids and antihistamine drugs were needed to continue the radiosurgical procedure to its end.

**Conclusion:**

Allergic reaction of the skin during radiosurgery with a thermoplastic mask for head fixation can develop due to cutaneous contact of the face with the mask.

## Background

Radiation therapy for head-and-neck tumors requires a reliable immobilization for an accurate and consistent treatment setup. Head immobilization with a thermoplastic face mask is the most frequently used method [1–4]. We report a case of skin allergy to a component of the thermoplastic mask made for a brain hypofractionated radiation treatment.

## Case presentation

**Fig. 1:**
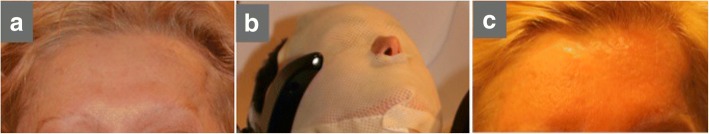
**a** Patient’s forehead before mask making. **b** Thermoplastic mask on the patient’s face during mask making. **c** Patient’s forehead after mask making, with acute allergic cutaneous reaction on the forehead and edema

A 71-year-old Caucasian woman was treated in our Gamma Knife center for a meningioma of the sphenoid jugum. The treatment was performed with Leksell Gamma Knife Icon® (Elekta Instruments, Stockholm, Sweden) and was planned as a hypofractionated irradiation including five daily fractions of 5 Gy. The restraint method chosen was the use of a thermoplastic mask Orfit® (Orfit Industries, Wijnegem, Belgium) [1, 4]. The mask was made 5 days before the first irradiation. During mask making, the mask was warmed by soaking in a water bath and then applied and molded directly on our patient’s face for 20 minutes (Fig. 1b). At this step of the procedure, she complained of a burning and tingling sensation on her face, especially on her forehead. During the following 4 days, she continued to have a stable cutaneous reaction in the form of redness, tickling, and edematous swelling of her face. She was treated with a local antihistamine cream on her face, with moderate improvement in the symptoms. On the first day of treatment, during the first irradiation session, she complained again of a major sensation of burning and edema of the face. A clinical examination showed a serious allergic reaction on her face, associated with an atopic edema. She was treated with 125 mg of intravenously administered corticoids, followed by high doses of orally administered antihistamines and corticoids during the following 5 days. With this medication, the allergic reaction was controlled until the end of treatment 4 days later. During all irradiation fractions we kept using the thermoplastic mask but we inserted a thin sheet of paper between the internal surface of the mask and our patient’s forehead to reduce the surface area of contact between the mask and our patient’s skin.

## Discussion

Thermoplastic masks are commonly used in radiation therapy to immobilize the head during irradiation [1, 4]. The mask used for our patient is recommended by Elekta for frameless treatments with the Leksell Gamma Knife Icon®. The procedure of mask making requires direct contact between the mask and the patient’s skin. An atopic reaction of the skin can occurs if a component of the mask is potentially allergenic. The main component of these masks is polycaprolactone (PCL), which is biodegradable polyester with the chemical formula (C_6_H_10_O_2_)*n*. PCL is prepared by polymerization of ε-caprolactone using a catalyst. The European Chemicals Agency provides Globally Harmonized System of Classification and Labelling of Chemicals (GHS) Hazard Statement information on PCL that notifies that PCL “may cause an allergic skin reaction (Warning Sensitization Skin - Category 1)”.

To the best of our knowledge, this is the first report to document the occurrence of an acute allergic reaction to the thermoplastic mask used for radiotherapy. We recommend starting a treatment with antihistamine and corticoids as soon as possible when a patient presents obvious allergic cutaneous reaction immediately after contact with thermoplastic mask during mask making. The interposition of a thin protection between skin and mask, such as a sheet of paper, could reduce the allergic reaction during treatment with the mask.

## Conclusions

Thermoplastic masks used for head immobilization during radiosurgery and radiotherapy procedures can induce an allergic reaction that may threaten the proper continuation of the irradiation therapy. Treatment of the allergic reaction with corticoids and antihistamine drugs is useful, as well as the interposition of a sheet of paper between the internal part of the mask and the skin.
